# Shh Gene Regulates the Proliferation and Apoptosis of Dermal Papilla Cells to Affect Its Differential Expression in Secondary Hair Follicle Growth Cycle of Cashmere Goats

**DOI:** 10.3390/ani14142049

**Published:** 2024-07-12

**Authors:** Junjie Zhang, Yujing Liu, Jiale Chang, Ru Zhang, Zhaomin Liu, Jiayue Liang, Dong Wang, Juan Feng, Wei Zhao, Hongmei Xiao

**Affiliations:** 1 College of Life Sciences, Inner Mongolia Agricultural University, Hohhot 010010, China; 2 Inner Mongolia Autonomous Region Key Laboratory of Biomanufacturing, Hohhot 010010, China; 3 Institute of Microbiology, Chinese Academy of Sciences, Beijing 100101, China

**Keywords:** cashmere goat, DPCs, Shh, proliferation, Hedgehog

## Abstract

**Simple Summary:**

Inner Mongolia Albas white cashmere goat is a unique biological resource in Northwest China, and the cashmere produced by goat is sold well at home and abroad because of its softness and warmth. Cashmere is the product of the growth and development of secondary hair follicles, and dermal papilla cells (DPCs) are the seeds of hair follicle growth and development, which are regulated by a variety of factors in growth and development. At present, the regulation mechanism of Shh gene on DPCs of secondary hair follicles in cashmere goats is not clear. In this study, we found that Shh gene expression was significantly higher in anagen than in catagen and telogen in the secondary hair follicles of cashmere goats, and the proliferation and viability of DPCs after overexpressing Shh gene were increased, whereas the apoptosis of DPCs after interfering with Shh gene was elevated. In addition to this, we demonstrated that Shh gene promotes the expression of the related genes Ptched (Ptch), Smoothened (Smo), and GLI Family Zinc Finger 2 (Gli2) in the Hedgehog signaling pathway. Therefore, this study reveals that Shh regulates the genes related to the Hedgehog signaling pathway to affect the proliferation and apoptosis of dermal papilla cells, leading to the differential expression of the Shh gene in the secondary hair follicle growth cycle of cashmere goats. The result is of great significance for the study of the pathway regulation mechanism of secondary hair follicles in cashmere goat and provides a theoretical direction for the positive development of the cashmere industry.

**Abstract:**

Sonic hedgehog (Shh) is a component of the Hedgehog signaling pathway, playing an important role in regulating cell proliferation, differentiation, apoptosis, and the repair of damaged organisms. To further clarify the expression pattern of Shh gene in the secondary hair follicle growth cycle of cashmere goats and its mechanism of action on secondary hair follicle papilla cells, and improve cashmere quality, in this study, we took Inner Mongolia Albas white cashmere goats as the research objects and collected skin samples at different growth stages to obtain secondary hair follicles, detected Shh and its gene expression by RT-qPCR, Western blot, immunohistochemistry, and other techniques, while we also cultured DPCs in vitro. Shh gene overexpression and interference vectors were constructed, and the effects of Shh gene on the proliferation and apoptosis of DPCs were studied through cell transfection technology. The results showed that there are significant differences in Shh and its gene expression in the secondary hair follicle growth cycle skins of cashmere goats, with the highest expression level in anagen, followed by catagen, and the lowest expression level in telogen. Shh was mainly expressed in the inner root sheath, outer root sheath, and secondary hair follicle papilla. After the overexpression of Shh gene, the proliferation and vitality of the hair papilla cells were enhanced compared to the interference group. After Shh gene interference, the apoptosis rate of the cells increased, indicating that Shh gene can regulate downstream Ptch, Smo, and Gli2 gene expression to promote the proliferation of DPCs, and thus form its expression pattern in the secondary hair follicle growth cycle of cashmere goats.

## 1. Introduction

Cashmere goat is a breed of goat with a distinctive fleece texture that originated in the Kashmir region near the Himalayas. In terms of biological classification, it belongs to *Mammalia*, *Bovidae*, *Caprinae*, and *Capra*. Cashmere is known as a brilliant gem in the fiber industry, with a quality far superior to other natural or artificial fibers, and extremely high economic value. Inland Asian countries are the main producers of cashmere, of which China has a wide variety and distribution of cashmere goats, each with its own characteristics and advantages. At present, both the quantity of cashmere goats and fiber production have jumped to the top in the world. Inner Mongolia Albas white cashmere goat is a major breed of cashmere goat in China, formed through natural selection and artificial breeding. It distributes in the cold semi-desert grasslands of Ordos in Inner Mongolia. Its high-quality fleece products have long brought great economic benefits for China’s textile industry, clothing industry, etc., and the famous “Ordos” cashmere products sell well at home and abroad [1,2]. In recent years, due to the change in breeding methods and the pursuit of production volume, the quality of fleece has decreased. Therefore, improving cashmere quality has become an important task in the study of cashmere goats.

Hair follicle (HF) is an appendage of cashmere goat skins, which can be divided into two types according to its developmental status, namely primary and secondary hair follicles [3]. Primary hair follicles are formed by the collaboration of epidermal and dermal cells, whereas secondary hair follicles grow out of primary follicles near the epidermis independently, forming a unique growth path that grows in clusters around the primary hair follicle and forming a group of hair follicles, that is, trichomes, which are distributed on the body surface. Due to the different growth paths, primary hair follicles produce long and thick hair, and secondary hair follicles produce thin and short fluff [4]. The difference in the quality of hair and fluff leads to the difference in economic value. Hair follicles are uniquely capable of self-renewal and cyclic growth [5]. Based on morphological changes, the hair follicle growth cycle was divided into anagen, catagen, and telogen. Each of these periods is tightly regulated and exhibits large differences in gene expression, cell proliferation, and cell differentiation [6,7]. Hair follicles consist of four parts: scapus pili, inner root sheath (IRS), outer root sheath (ORS), and hair bulb. The secondary hair follicles of cashmere goat are characterized in the same way. Hair papilla is the inwardly concave part of the base of the hair bulb, and hair papilla cells have certain heterogeneity and characteristics of adult stem cells, which are also known as dermal papilla cells (DPCs). As the core of regulatory signaling, DPCs release a variety of signaling molecules through paracrine and other mechanisms, guiding the surrounding epidermal cells, i.e., stromal cells, to divide and proliferate, migrate upward, and differentiate into the scapus pili, inner root sheath, and outer root sheath structure [8,9], and then regulate the cyclical changes in hair follicle growth and maintain and promote the healthy growth of hair follicles [10].

However, it is not clearly known which factors play an essential role in DPCs’ proliferation and development.

The signaling pathways involved in the cyclic growth of secondary hair follicles include Wnt, Hedgehog, BMP, Notch, etc. The Hedgehog signaling pathway controls embryonic development, which has a significant impact on cell survival, growth, and differentiation after embryonic development and embryonic cell formation, and it plays a crucial role in embryonic development and the maintenance of homeostasis in adult tissues. Hedgehog mainly consists of three homologous gene ligands, Sonic Hedgehog (Shh), Indian Hedgehog (Ihh), and Desert Hedgehog (Dhh), two receptor proteins Patched (Ptch), Smoothened (Smo), Suppressor of Fused (SuFu), G protein-coupled receptor kinase (GRKs), β-arrestins, and downstream transcription factor GLI [11,12,13]. The transduction of its signaling is mainly mediated by transmembrane proteins Ptch1 and Smo. When Shh is not present, Ptch1 binds to Smo and inhibits the activity of Smo, leading to a complex formation between Gli and SuFu in the cytoplasm, which is hydrolyzed by the proteasome and inhibits the transcription of its target gene. Conversely, when Shh is present, Shh binds to Ptch1, relieving its inhibitory effect on Smo; activated Smo dissociates the SuFu-Gli complex, which leads to the entry of Gli into the nucleus and facilitates the transcription of the target gene [14,15,16]. The aberrant activation of the Hedgehog pathway has been associated with many cancers [17,18,19], such as brain, liver, lung gastric, breast, and hematologic malignancies. In addition, the Hedgehog signaling pathway is also involved in a variety of physiological processes, such as T cell development and differentiation [20,21,22,23], gastrointestinal inflammatory response [24,25,26], adipose inflammatory response [27,28], and regulation of the nervous system [29,30,31,32,33].

Studies have found that Shh has a wide range of functions in organisms, not only related to cancers such as rectal cancer, pancreatic cancer, and breast cancer, or embryonic development diseases [34], but also substances regulating hair follicles and hair growth, such as growth factor (TGF-β), alkaline phosphatase (ALP), and fibroblast growth factor (FGF), and so on [7,35]. For example, after Shh knockout or interference, the development of a hair follicle germ in mice is hindered, and hair follicle structure cannot be formed as expected, which ultimately affects hair growth [36]. Therefore, it is of great significance to study the function and mechanism of Shh gene in the development of organisms in depth.

In this study, we revealed the molecular and cellular levels that affect Shh gene and its expression in the hair follicle growth cycle by influencing the proliferation and apoptosis of DPCs in the secondary hair follicles of Inner Mongolia Albas white cashmere goats. These results will provide a molecular theoretical basis for regulating the growth and development cycle of cashmere and improving the quality of cashmere.

## 2. Materials and Methods

### 2.1. Animal and Sample Collection

The experimental animals were three healthy 1-year-old Inner Mongolia Albas white cashmere goats. A total of 1 cm^2^ of skin was collected from behind the ear and from the side of the body at a distance of about 25 cm from the scapula, and the skin was collected once at the telogen (February), anagen (September), and catagen (December) of the secondary hair follicles. The postauricular skin tissue was washed with 75% alcohol for 15 s and then washed three times with phosphate-buffered saline (PBS) containing 1% penicillin bispecific antibody solution to remove the connective tissue of the skin and then cut into three pieces of the tissue in the direction of hair growth and placed in DMEM/F-12 containing 2% penicillin bispecific antibody solution for spare parts. Body side skin tissue samples were rapidly stored in liquid nitrogen.

### 2.2. In Vitro Culture of DPCs

A single secondary hair follicle in anagen was separated from the skin tissue digested with 0.25% neutral protease using a dissecting needle, and the hair papilla part cut was cultured in a cell culture dish (DMEM/F-12 containing 1% bispecific antibody and 15% fetal bovine serum), and the culture conditions were 5% CO_2_ concentration, saturation humidity 37 °C, and the culture was static for 5–7 days. After the cell growth density was 80%, monoclonal multiplication and subculture were carried out. We observed the cell status at regular intervals and changed the medium. The specific marker genes α-SMA and CD133 were identified by immunofluorescence detection. All invitro breeding was carried out from one resident.

### 2.3. Real-Time Quantitative PCR (RT-qPCR)

Primers were designed by Primer 5.0 software based on the CDS sequences of Shh gene, PCNA, Ki67, Ptch, Smo, and Gli2 genes of cashmere goats provided by NCBI (https://www.ncbi.nlm.nih.gov/ (accessed on 17 September 2023)) (Table 1). The total RNA of the skins and DPCs from the secondary hair follicles in different growth stages extracted using TRIzon Reagent (CWBIO, Taizhou, Jiangsu, China) was used to synthesize the first strand of cDNA by PCR, and the reaction conditions were incubated at 37 °C for 15 min, heated at 85 °C for 5 s, and cooled at 4 °C. Reaction system: 1 μg of RNA, 2 μL of 5× RT Buffer, and aseptic enzyme-free water were made up to 10 μL. The relative expression of Shh, PCNA, Ki67, Ptch, Smo, and Gli2 genes in hair follicle growth cycles and DPCs was detected by RT-qPCR with the reaction system configured according to the TB Green Premix Ex Taq II (Tli RNaseH Plus) (TaKaRa, Dalian, China) reagent instructions. Reaction system: Template 1 μL, Forward Primer 1 μL, Reverse Primer 1 μL, Master Mix 10 μL, enzyme-free water 7 μL.

### 2.4. Western Blot

The total proteins extracted from the skins at different growth stages of the secondary hair follicles of cashmere goats were quantified by bicinchoninic acid (BCA) and then diluted in concentration, and a certain amount of Loading buffer (5×) was added and boiled at 100 °C for 5 min to achieve protein denaturation, and the proteins were transferred to the PVDF membrane after electrophoretic separation and then placed on the shaker at room temperature in the blocking solution (5% skimmed milk powder) for 1~3 h. We diluted the Shh and GAPDH antibodies and incubated them on a shaker at room temperature for 1 h, and then they were placed at 4 °C to incubate overnight. The following day, we washed with TBST on a shaker at room temperature before adding secondary antibody (1:5000) and incubated at room temperature for 2 h. After washing with TBST, we used a fluorescence imaging solution and performed imaging operations in the gel imaging equipment (ImageQuant LAS 500, GE Healthcare, Piscataway, NJ, USA).

### 2.5. Immunohistochemistry

The prepared paraffin sections of skins were put into xylene and sequentially soaked in 70%, 80%, 95%, and 100% ethanol for 8 min, soaked and washed in distilled water for 5 min, and then immersed in citrate buffer, heated in the microwave oven for 8 min, and subsequently cooled down to room temperature. PBS was washed and treated with 3% hydrogen peroxide for 10 min, and the washed sections were incubated in 5% sheep serum for 20 min at room temperature and dropwise added with a primary antibody of the appropriate concentration. A control group was set up and stained with PBS instead of the primary antibody, incubated overnight at 4 °C, subsequently rewarmed and washed in light, avoiding rewarming, before adding secondary antibody dropwise and incubating for 30 min. DAB chromogenic working solution was added dropwise, incubated at room temperature for 10 min, hematoxylin counterstained for 8 min, we rinsed the distilled water, the PBS returned to blue, the alcohol gradient was dehydrated (70%, 80%, 95%, 100% alcohol) for 5 min each, xylene transparent occurred twice, neutral gum mounting occurred, they were dried overnight, and then we observed them under a fluorescence inverted microscope (Eclipse Ti2-U, Nikon, Tokyo, Japan).

### 2.6. Transformation and Extraction of Expression Vectors and Recombinant Products

The overexpression vector GV102 (+) and the interference vector GV658 (−) were synthesized by Shanghai Jikai Gene Technology Co., Ltd. (Shanghai, China).

Competent cell DH5α was added to the bacterial solution after 10 min of ice bath and mixed gently (diluted 50/20/non-diluted), followed by ice bath for 30 min, placed in a water bath for heat shock, and left on ice for 2 min; subsequently, we added antibiotic-free liquid medium, mixed well, and then resuscitated the bacterial. The bacterial solution was spread on the plate containing LB solid medium and incubated at 37 °C for 12–16 h. We picked single colonies and cultured them in liquid medium containing ampicillin for another 12–16 h and then extracted the plasmids from the bacterial fluid and performed enzymatic digestion using restriction endonucleases KpnI and PcnI. The plasmids verified by double digestion were sent to Bio for sequencing.

### 2.7. DPCs Transfection and Viability Detection

DPCs cultured in vitro were seeded into 24-well plates until the cell density reached 70%. The medium was then replaced with DMEM (Gibco, Thermo Fisher Scientific, Suzhou, China), and after 6 h, the cells were transfected using Lipofectamine 2000 (Invitrogen, Carlsbad, CA, USA). The transfection groups included the following: Shh overexpression group (ov-Shh) and overexpression control (ov-control), as well as Shh interference group (si-Shh) and interference control group (normal cells, NC), with three replicates each. Twenty-four hours post-transfection, DPCs were evenly distributed in a 96-well plate, and CCK8 solution was added and incubated in the incubator for 1–4 h. Absorbance at 450 nm was measured using a Microplate Reader (FlexStation 3, Molecular Devices, San Jose, CA, USA) to calculate the relative cell viability.

### 2.8. Apoptosis Detection of DPCs

After transfection, DPCs were centrifuged at 1000× *g* for 5 min to collect the cell pellet. The cells were then resuspended in Annexin V-FITC binding solution (Beyotime, Shanghai, China), and both Annexin V-FITC and propidium iodide were added and mixed well. The mixture was incubated at room temperature in the dark for 10–20 min before being placed on ice. Subsequently, apoptotic cells were evaluated using a NovoCyte Flow Cytometer (ACEA Biosciences Inc., San Diego, CA, USA) equipped with NovoExpress 1.5.0.

### 2.9. Data Statistical Analysis

The results were analyzed using GraphPad Prism (version 9.5.0). The relative expression levels of Shh, Ki67 and PCNA genes, and genes related to the Hedgehog signaling pathway, Ptch, Smo, and Gli2, were calculated using 2^−ΔΔCt^. Student’s *t*-test was used for comparison between the two groups, and Turkey’s test was used for detecting differences between the three groups. The obtained *p*-values < 0.05, indicated by “*”; *p* < 0.01, indicated by “**”; *p* < 0.001, indicated by “***”; *p* < 0.0001, indicated by “****”, indicating that the differences were statistically significant.

## 3. Results

### 3.1. Expression of Shh Gene in Secondary Hair Follicle of Cashmere Goats

#### 3.1.1. Expression of Shh Gene in Secondary Hair Follicle Cycle Growth Skins

**Figure 1:**
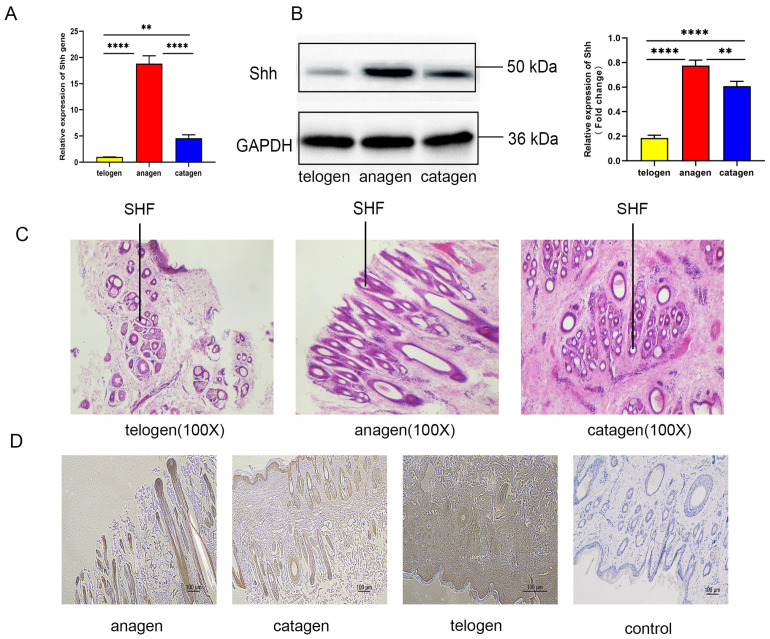
Expression of Shh and its genes: (**A**) Shh gene expression in different stage skins of secondary hair follicle growth; (**B**) Shh expression in different growth stag skins of secondary hair follicles; (**C**) H&E staining of hair follicle slices at different growth stages; (**D**) Shh expression site in secondary hair follicles. “**” and “****” indicate *p* < 0.01 and *p* < 0.0001, and their differences are statistically significant.

As shown in the RT-qPCR detection results (Figure 1A), the expression level of Shh gene is the highest in anagen, followed by catagen, and the lowest level in telogen. This indicates that Shh gene expression in growth cycle skins is consistent with the trend in secondary hair follicle growth cycles in cashmere goats.

#### 3.1.2. Expression of Shh in Secondary Hair Follicle Cycle Growth Skins of Cashmere Goats

The results of the Western blot experiment are shown in Figure 1B. The expression level of Shh in the secondary hair follicle growth cycle skins is consistent with the RT-qPCR experiment results. The expression level is highest during anagen, followed by catagen, and lowest during telogen.

#### 3.1.3. Expression Site of Shh in Secondary Hair Follicles

The different stage skin tissue sections with clear and intact outlines of primary hair follicles and secondary hair follicles (SHFs) observed in the hematoxylin and eosin (H&E) staining skin tissue sections from Inner Mongolia Albas white cashmere goats (Figure 1C) are selected to detect the expression sites of Shh in secondary hair follicles by immunohistochemistry technology. And the results showed (Figure 1D) that Shh is expressed at different growth stages of secondary hair follicles, with varying levels of expression. The expression level is highest in anagen and lowest in telogen. Shh is expressed in the outer root sheath, inner root sheath, and hair papilla during the growth period of secondary hair follicles, indicating that Shh has a promoting effect on the development of secondary hair follicles in cashmere goats.

### 3.2. Effect of Shh Gene Overexpression on Proliferation and Apoptosis of Secondary Hair Follicle DPCs in Cashmere Goats

#### 3.2.1. DPCs Cultivation in vitro and Identification

**Figure 2:**
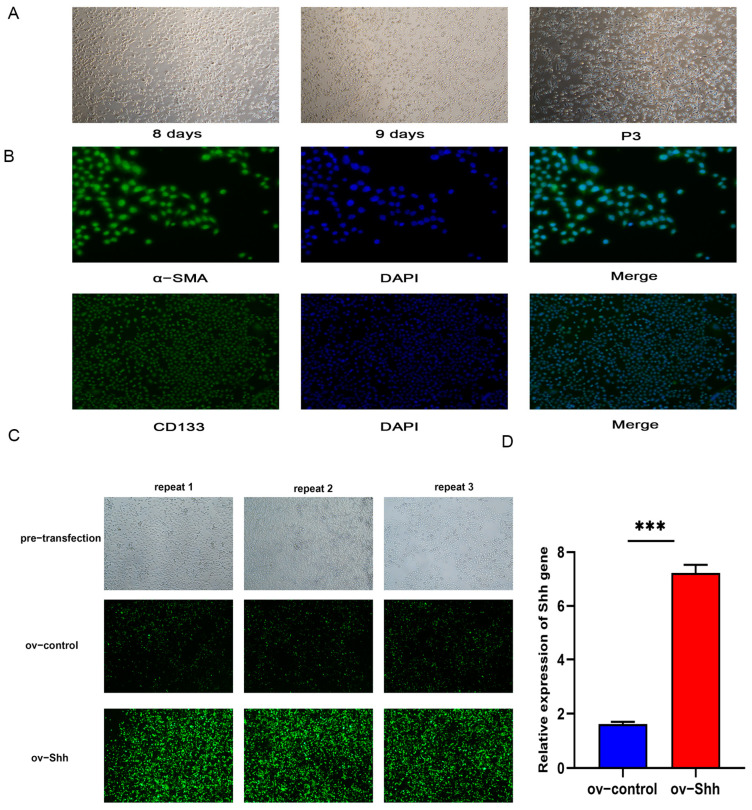
Identification of DPCs and construction of overexpression vectors: (**A**) primary and passage culture of DPCs; (**B**) immunofluorescence identification of DPCs by markers α-SMA and CD133, DAPI is the nucleus, merge is a mixture of antibodies and cell nucleus; (**C**,**D**) detection of overexpression vector transfecting DPCs. “***” indicates *p* < 0.001, indicating statistical significance compared to overexpression controls.

On the basis of in vitro primary culture, the growth rate of DPCs was accelerated after passage culture. The cell morphology is often polygonal or irregular diamond-shaped (Figure 2A). To ensure that the cultured cells are DPCs, the isolated DPCs were detected using specific marker genes α-SMA and CD133. Immunofluorescence staining showed positive results, indicating that the cultured cells were papillary cells of secondary hair follicles (Figure 2B).

#### 3.2.2. Construction of Shh Gene Overexpression Vector and DPCs Transfection

Using the transfection method of Lipofectamine 2000 (Invitrogen, Carlsbad, CA, USA), the overexpression plasmid and overexpression empty plasmid of Shh gene were transfected in DPCs 24 h with the transfection reagent (Figure 2C). GFP carried by the transient transfection vector was transferred into DPCs and showed green fluorescence under the fluorescence microscope, indicating successful transfection. Moreover, the green fluorescence in the overexpression group was significantly higher than that in the control group, indicating that the transfection efficiency of the experimental group was higher than that of the control group (Figure 2D).

#### 3.2.3. Detection of DPCs’ Proliferation and Apoptosis

**Figure 3:**
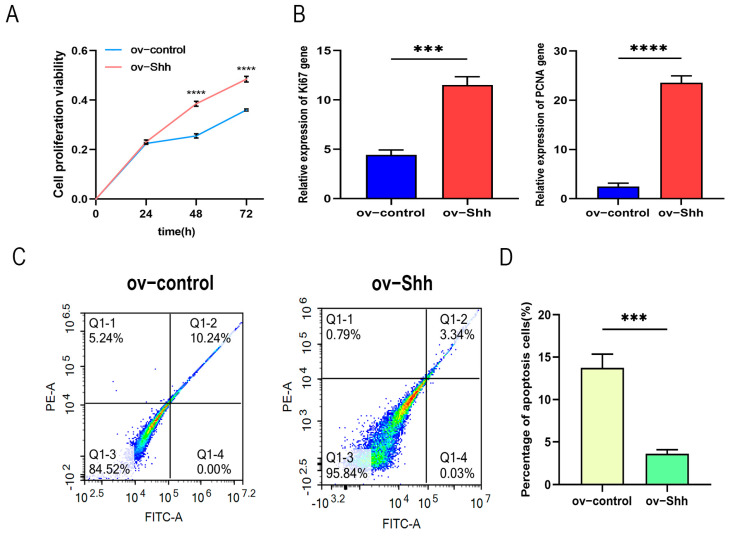
Proliferation and apoptosis assay of DPCs overexpressing Shh: (**A**) detection of CCK8 proliferation activity; (**B**) Ki67 and PCNA genes expression in DPCs; (**C**) cell apoptosis detected by flow cytometry; (**D**) analysis of cell apoptosis rate. “***” and “****” indicate *p* < 0.001 and *p* < 0.0001, respectively, indicating that the differences are statistically significant.

Using CCK8 to detect the proliferation of DPCs transfected for 24 h, 48 h, and 72 h, the cell proliferation curve was obtained by measuring the absorbance value at 450 nm. Compared with the control group, the proliferation rate of the cells significantly increased at 48 h (Figure 3A).

To verify the effect of the overexpression of Shh gene on the DPCs’ proliferation ability, RT-qPCR was used to detect the expression of proliferation-related PCNA and Ki67 genes (Figure 3B). It was found that the expression levels of Ki67 and PCNA genes remarkably rose after Shh gene overexpression. The overexpression of Shh gene promotes the proliferation of DPCs.

The live cell ratio, early apoptotic cell ratio, late apoptotic cell ratio, and necrosis rate of DPCs treated with the Annexin V-FITC/PI dual staining cell apoptosis kit were detected by flow cytometry, as shown in Figure 3C,D. The normal cell survival rate in the control group cells was 84.52%, the survival rate of the cells in the overexpression group of Shh gene was 95.84%, and the apoptosis rate of the cells in the overexpression group was significantly lower than that in the control group, indicating that the overexpression of Shh gene can inhibit DPCs apoptosis and reduce the apoptosis rate of cells.

### 3.3. Effect of Interfering with Shh Gene on the Proliferation and Apoptosis of Secondary DPCs in Cashmere Goats

#### 3.3.1. Construction of Shh Gene Interference Vector and DPCs Transfection

**Figure 4:**
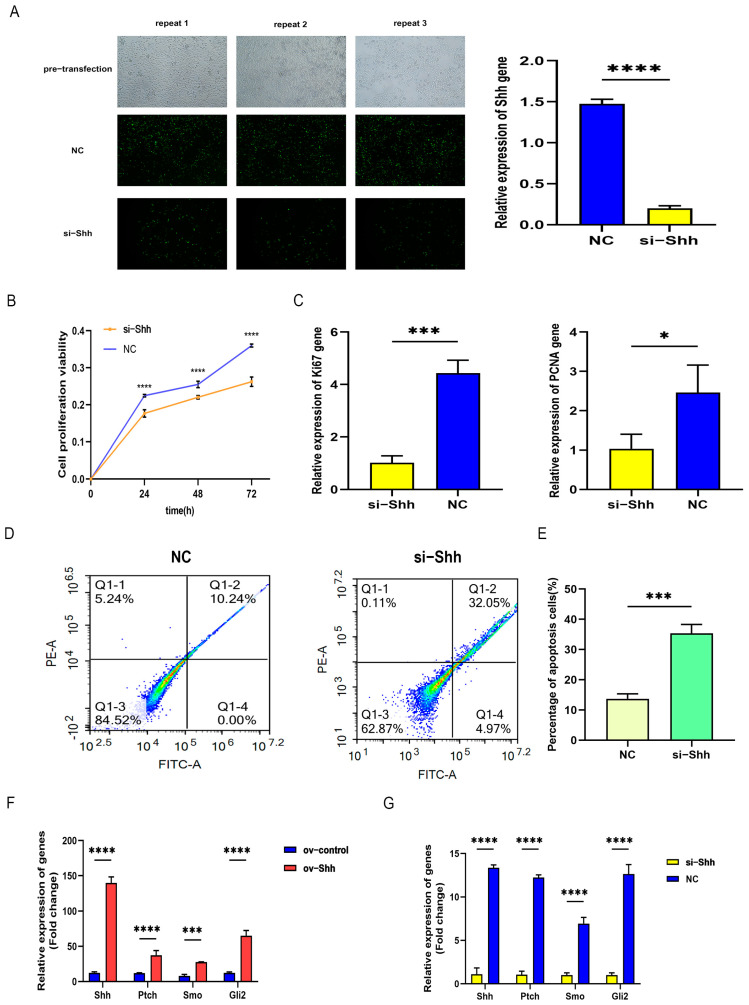
Construction of Shh interference vector and apoptosis detection of DPCs: (**A**) detection of interference vector transfecting DPCs; (**B**) the proliferative activity of DPCs assayed by CCK8; (**C**) Ki67 and PCNA genes expression in DPCs; (**D**) cell apoptosis detected by flow cytometry; (**E**) analysis of cell apoptosis rate; (**F**,**G**) the expression levels of Ptch, Smo, and Gli2 in DPCs overexpressing and interfering with Shh were detected by RT-qPCR. “*”, “***”, and “****” indicate *p* < 0.05, *p* < 0.001, and *p* < 0.0001, respectively, and their differences are statistically significant.

The interference vector sequence of Shh gene was designed and constructed by Shanghai Jikai Gene Company, with specific information shown in Table 2. Using the transfection method of Lipo2000 reagent, the Shh gene interference plasmid and interference empty plasmid were transfected into DPCs for 24 h (Figure 4A), DPCs of both the NC group and interference group showed green fluorescence, and the number of green DPCs in the NC group was significantly higher than that in the interference group, indicating successful interference. The interference efficiency of the interference group was higher than that of the NC group.

#### 3.3.2. Proliferation and Apoptosis Detection of DPCs

CCK8 was used to observe the proliferation of DPCs transfected for 24 h, 48 h, and 72 h (Figure 4B); the results showed that DPCs’ proliferation activity of the interference group was lower than that of the NC group over time, and there was a significant difference between the interference group and NC group after 24 h of transfection, revealing that interference with Shh gene can reduce DPCs’ proliferation activity.

RT-qPCR was used to detect the expression of proliferation-related genes’ PCNA and Ki67, as shown in Figure 4C. After interfering with Shh gene, the expression levels of Ki67 and PCNA genes were significantly reduced. This indicates that the proliferation of DPCs is inhibited through interfering with the Shh gene.

The apoptosis of transfected DPCs was detected by flow cytometry, as shown in Figure 4D. The normal cell survival rate of the NC group was 84.52%, while the survival rate of interference group cells was significantly reduced to 62.87%, indicating that interference with Shh gene promotes the apoptosis of DPCs (Figure 4E).

### 3.4. Effect of Shh Gene on the Expression of Hedgehog-Signaling-Pathway-Related Genes

By RT-qPCR, the relative expression levels of Ptch, Smo, and Gli2 genes as the Shh receptor were detected in DPCs transfected of the Shh gene overexpression group, interference group, overexpression control, and interference control. The test results displayed that the expression levels of the three genes were significantly higher in the overexpression group than that in the control group (Figure 4F); in the interference group, their expression was significantly lower than that in the control group (Figure 4G). It manifests that Shh gene can promote the expression of Ptch, Smo, and Gli2 genes.

## 4. Discussion

As the regulatory center of entire hair follicle activity, DPCs send signals to act on hair matrix cells, stimulating their proliferation and differentiation, and regulating the regeneration and maintenance of hair follicles [37,38]. DPCs are not only essential for inducing and maintaining the cyclical cycle of hair follicle growth but their size also determines the size of hair follicles and the length of their growth period.

Previous studies have found that the growth and development of hair follicles and DPCs rely on the joint regulation of various growth factors, small molecule compounds, trace elements, and other nutrients, as well as various complex network signaling pathways. The signal pathways mainly include the classic Wnt, Eda/Edar, BMP, TGF-β, PI3K/Akt, etc. Growth factors include Wnt10, The VEGF family, FGf20, IGF, IGF-BP, BMP4, HGF, ALP, Vertical, KGF, AR, and so on. Nutrients include various elements and compounds such as vitamin D, vitamin A, melatonin [39], exosomes, methionine, cysteine, and androgens. Shh signaling regulates the survival and death of hair follicle stem cells (HFSCs) by cross-talking with signaling pathways, such as the Wnt/β-catenin signaling pathway, BMP, and others [35]. Suzuki et al. [40] obtained similar conclusions when studying hair follicles in mice. Noggin, an antagonist of BMP, ameliorates the stagnation of hair follicle development caused by the knockdown of dermal Smo by increasing epithelial Shh expression [41]. The lack of riboflavin can lead to hair loss [42], B vitamins may promote the growth and development of hair follicles, while androgens have an inhibitory effect on hair follicle growth.

Moreover, these growth factors and substances jointly mediate the signal exchange between epidermal and dermal parts of hair follicles through autocrine, paracrine, or external acquisition, promoting the proliferation and differentiation of hair follicle cells [43,44], thereby regulating the cyclical changes in hair follicle growth [45,46].

The Hedgehog signaling regulated by Shh plays an important role in the occurrence and regeneration of almost all organs of mammals [47,48]. Studies have shown that after injecting Shh into the back of nude mice for 2 weeks, it was found that the back hairs in the injection group were longer and denser than those of the control group and actually grew faster, with an increase in tyrosinase and hair-specific keratin in the hair follicles [49]. In tabby mice, if the Shh gene is knocked-out, the mice appear hairless, able to form primary and secondary hair follicle germs, but unable to develop further. When the Shh of the skin is lost, the secondary hair follicle germ starts normally but cannot develop further [50]. The offspring of pregnant mice treated with anti-hedgehog blocking monoclonal antibody showed hairless characteristics. The inhibition of body hair morphogenesis was also observed in postnatal mice during the hair growth phase using anti-hedgehog monoclonal antibodies [51]. Adult hair follicle regeneration is associated with Shh secretion by HFSCs progeny as well [52]. All these indicate that the Shh gene is necessary for the complete development of hair follicles [53,54,55,56]. In this research, overexpression and RNAi techniques [57] were used to regulate the expression level of Shh gene in the secondary hair papilla cells of cashmere goats. The proliferation and apoptosis of hair papilla cells were detected using CCK8 and flow cytometry to explore the effect of Shh gene on the secondary hair papilla cells of cashmere goats. The results indicate that Shh gene has a positive regulatory effect on the proliferation of DPCs in the secondary hair follicles of cashmere goats by regulating the downstream gene Ptch, Smo, and Gli2 expression to promote the growth and development of hair papilla cells. These results will provide a positive molecular theoretical basis for the breeding of the cashmere goat and the development of the cashmere industry. However, the regulatory mechanism of Shh gene on the secondary hair follicle of cashmere goat has not been clearly analyzed yet, and further research and verification are needed. In order to gain a deeper understanding of the regulatory mechanism of the Shh signaling pathway in cashmere growth and its relationship with other signaling pathways, experimental research will be conducted on Gli2 target gene identification, gene editing, protein interaction, and other aspects.

## 5. Conclusions

Our study demonstrates that the Shh gene is able to regulate the proliferation and apoptosis of DPCs, thereby affecting its differential expression during the growth cycle of secondary hair follicles in cashmere goats. This will provide a positive impact on the cashmere industry in terms of regulating the growth and apoptosis of secondary hair follicles in cashmere goats at the molecular level.

## Figures and Tables

**Table 1:** The primer sequences.

Gene	Primer Sequence (5′-3′)
Shh	F: AGCCTACAAGCAGTTTATCCC R: GGTCCGCTCCAGTGTTTTC
PCNA	F: AGAGGAGGAAGCTGTTACCAT R: GACAGTGGAGTGGCTTTTGT
Ki67	F: TGTTGCCAAAATAGCTGCTG R: GTACCGTTTCACTGCTGGAT
Ptch	F: AGGCAGCGGTAGTAGTAGTG R: GTAGCGGGTATTGTCCGTG
Smo	F: CAACCCTCTGGGTCTTCCCTACT R: CGCTTCGTCTTCTGGCTGCTC
Gli2	F: GGTAGCTGGCTGATCCGAATTG R: TACACTGCGGCTCTGAACACT

**Table 2:** Sequences of siRNA-Shh and siRNA-NC.

Gene	Name	Sequence 5′→3′	Size (bp)
siRNA-Shh	a	GATCCCCTCGAGTTTTTGGAT	21
	b	AGCTATCCAAAAACTCGAGGG	21
siRNA-NC	a	GCACATCCACTGCTCAGTGAA	21
	b	AGCTATCCAAAAACTCGAGGG	21

## Data Availability

The data presented in this study are not publicly available and can be obtained from the corresponding author.
